# Supplementary material for: Different Responses of Bacteria and Archaea to Environmental Variables in Brines of the Mahai Potash Mine, Qinghai-Tibet Plateau
Source: Microorganisms. 2023 Aug 3;11(8):2002. doi: 10.3390/microorganisms11082002 (PMC10458105; doi:10.3390/microorganisms11082002)
Supplement: Supplementary file 1 [file microorganisms-11-02002-s001.zip › microorganisms-2453327-supplementary.pdf]

Table S1. Primers used in the amplification of the amplicons.

Primer name	Predicted target group	Target region	Primer sequences（5’→3’）
515F	Bacterial 16S rRNA	16S (V4+V5)	GTGCCAGCMGCCGCGGTAA
907R			CCGTCAATTCCTTTGAGTTT
Arch519F	Archaeal 16S rRNA	16S (V4+V5)	CAGCCGCCGCGGTAA
Arch915R			GTGCTCCCCCGCCAATTCCT

Table S2. Ionic chromatography analysis of brine samples from the Mahai potash area.

Sample ID	Anion concentrations (mg/L)				Cation concentrations (mg/L)				
	F^-	Cl^-	NO_3^-	SO_4^{2-}	Li^+	Na^+	K^+	Mg^{2+}	Ca^{2+}
**ML**	15003.43	4276.634	14849.78	15388.2	0	1752.437	1156.231	2951.274	3733.006
**MSa**	15008.67	157422.2	14876.42	21660.88	1048.775	68474.23	6127.609	17129.66	3958.655
**MSb**	15007.13	143291.8	14863.43	19374.23	1048.055	57633.01	5589.147	14234.05	4108.269
**MWa1**	14962.57	183747.7	15534.06	16970.09	1076.868	58005.92	8780.301	19554.33	4054.31
**MWa2**	14955.03	172267.5	15829.08	16849.51	1049.496	55424.79	1176.33	18605.3	4034.688
**MWa3**	14962.95	168074.3	15391.88	16547.81	1049.496	58640.92	8049.893	18693.78	4024.877
**MWa4**	14959.25	192179.7	15489.77	17130.95	1048.055	82309.46	2485.351	7152.701	3936.58
**MWb1**	14959.25	180899.7	15684.24	16003.92	1055.979	47078.67	9141.08	29525.59	4145.06
**MWb2**	14957.33	175603.9	15531.06	15986.73	1047.335	92249.42	1932.177	4700.917	4120.533
**MWb3**	14960.27	179258.3	15376.22	16023.42	1046.615	88844.11	1864.635	4548.216	4248.073
**MWb4**	14961.03	138688.1	15445.49	16619.9	1046.615	90848.92	1898.611	4750.866	4113.174
**MWc1**	14959.89	205499.2	15479.12	17161.22	1049.496	48882.24	2747.478	13967.18	4005.256
**MWc2**	14961.93	203283.7	15533.39	16996.01	1057.419	39564.63	23382.91	32715.19	5032.936
**MWc3**	14963.08	215179.1	15704.88	16861.83	1059.58	27149.8	18638.06	39640.98	4652.768
**MWc4**	14967.29	222592	15613.64	16735.35	1062.461	21301.77	20484.29	46138.63	4319.201
**MWc5**	14961.67	227679.6	15637.95	16687.12	1079.029	17609.27	19754.47	48784.51	3845.83
**MWd1**	14958.61	172371.6	14819.47	16569.36	1046.615	83249.3	2458.557	7272.579	3880.168
**MWd2**	14989.38	176794.6	14877.41	16812.57	1046.615	92986.42	2671.437	7866.259	3858.094
**MWd3**	14989.89	175264.2	14877.08	16769.21	1046.615	91957.87	2638.044	7830.581	3855.641
**MWd4**	-	-	-	-	-	-	-	-	-
**MWe1**	14958.61	175545.6	15335.27	17437.27	1047.335	83295.61	3731.664	8972.273	3811.493
**MWe2**	14961.29	187086.4	15415.52	17260.77	1056.699	36162.6	4862.85	35325.38	4412.404
**MWe3**	14960.27	189577.6	15457.14	16784.35	1064.622	9152.159	2913.536	50606.93	4221.093
**MWe4**	14960.14	213275.8	15428.17	17000.88	1067.504	13039.69	3201.55	55633.23	4417.309

“-”: data not aquired

Table S3. Sequence data of the 24 water samples.

Sample ID	No. of effective sequence		No. of assigned ASVs	
	Bacteria	Archaea	Bacteria	Archaea
**ML**	76051	104	727	34
**MSa**	74985	14020	640	60
**MSb**	74803	17732	1211	60
**MWa1**	75970	n.d.	1515	n.d.
**MWa2**	75853	20893	766	114
**MWa3**	75403	33230	1379	143
**MWa4**	74458	1487	1294	79
**MWb1**	76061	2044	924	10
**MWb2**	75936	737	1215	42
**MWb3**	75957	2365	759	47
**MWb4**	76001	10	886	6
**MWc1**	75673	1827	1220	73
**MWc2**	76032	37199	476	128
**MWc3**	75735	n.d.	2132	n.d.
**MWc4**	75984	12345	1688	77
**MWc5**	75917	5308	689	73
**MWd1**	74902	46018	875	104
**MWd2**	74578	40810	677	99
**MWd3**	75250	4464	694	64
**MWd4**	75498	162	734	43
**MWe1**	51246	14839	138	38
**MWe2**	75686	3978	2308	12
**MWe3**	75965	48	2014	12
**MWe4**	75906	92	96	5
**Total**	1789850	259712	3980	238

“n.d.”: not determined.

Table S4. Alpha diversity indices calculated for each sample.

Sample ID	Bacteria				Archaea			
	Richness	Chao1	Shannon	Invsimpson	Richness	Chao1	Shannon	Invsimpson
**ML**	727	1092.956	2.932158	5.582196	34	45.375	3.128652	16.09524
**MSa**	640	953.5119	2.803248	3.675312	60	61.5	3.053482	17.4974
**MSb**	1211	1639.879	3.395878	4.133344	60	77.5	3.112409	16.87359
**MWa1**	1515	1990.02	1.896605	2.047005	n.d.	n.d.	n.d.	n.d.
**MWa2**	766	1197.462	1.680828	2.319119	114	124.0435	1.654676	3.993343
**MWa3**	1379	1826.857	2.164484	2.310101	143	151.6364	2.058047	3.600064
**MWa4**	1294	1753.722	2.213416	2.393925	79	92.125	2.563019	5.445116
**MWb1**	924	1438.857	1.715922	2.446089	10	10.5	0.736642	2.011404
**MWb2**	1215	1682.708	2.813704	4.689674	42	49.33333	1.6573	2.860305
**MWb3**	759	1155.409	1.655671	1.963726	47	57.90909	1.873157	4.541625
**MWb4**	886	1281.189	1.93874	2.901035	6	7	1.695743	5
**MWc1**	1220	1623.077	3.101065	3.885334	73	108.4286	2.48619	7.669186
**MWc2**	476	799.0182	0.756326	1.232442	128	139.4	1.895657	3.497383
**MWc3**	2132	2502.722	3.968302	5.415725	n.d.	n.d.	n.d.	n.d.
**MWc4**	1688	2038.833	3.324904	5.010013	77	98.66667	2.108946	4.907463
**MWc5**	689	1084.116	1.871529	2.279554	73	90.76923	1.832427	4.431009
**MWd1**	875	1218.994	2.616186	4.732894	104	107.9286	3.391756	24.19337
**MWd2**	677	975.8095	2.010944	2.499024	99	108.5455	3.508113	27.84231
**MWd3**	694	1001.616	2.278593	2.298387	64	89	1.304112	2.641113
**MWd4**	734	1118.57	2.511628	2.516322	43	54.375	3.419129	23.18375
**MWe1**	138	159	3.915377	16.13991	38	38	2.81223	11.06877
**MWe2**	2308	2591.53	4.24077	7.103093	12	13.5	1.672916	4.828518
**MWe3**	2014	2379.889	4.099533	8.00471	12	33	1.995844	5.788945
**MWe4**	96	129	1.746239	2.073854	5	6	0.470767	1.252071

“n.d.”: not determined.

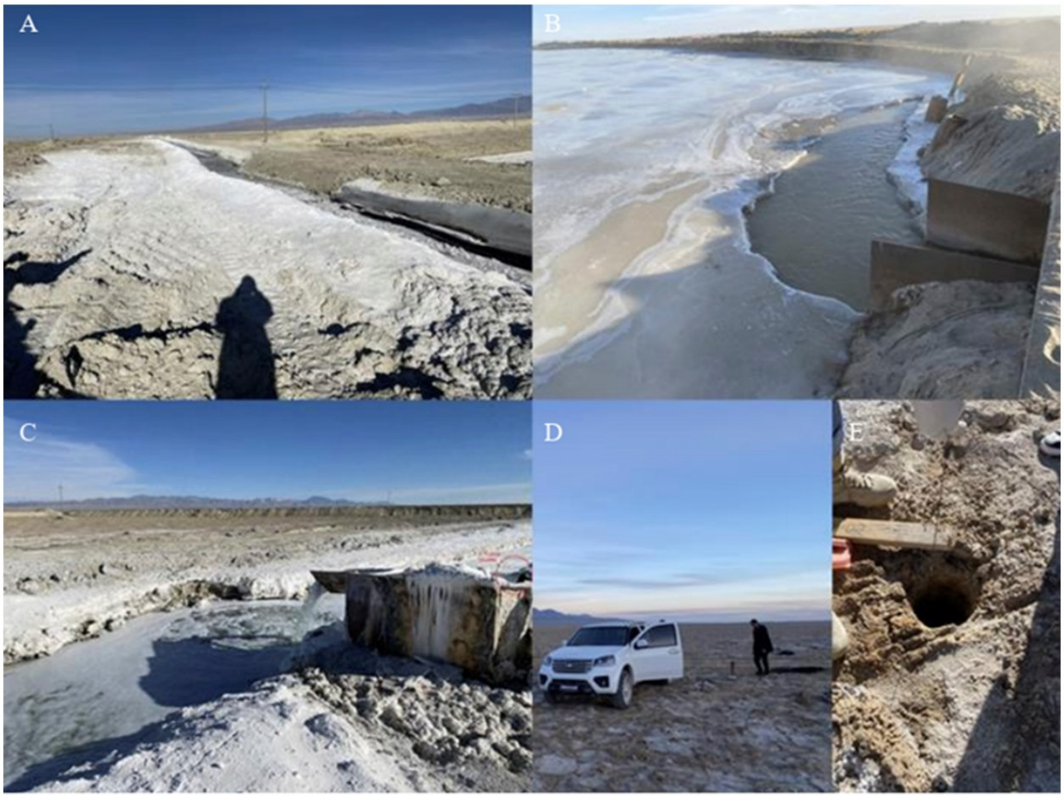

Figure S1. Sampling context. (A) The panorama of the Mahai potash area. (B) Mahai lake. (C) Monitoring station. (D) Sampling site of a Mining well. (E) Brine sampling from a mining well.

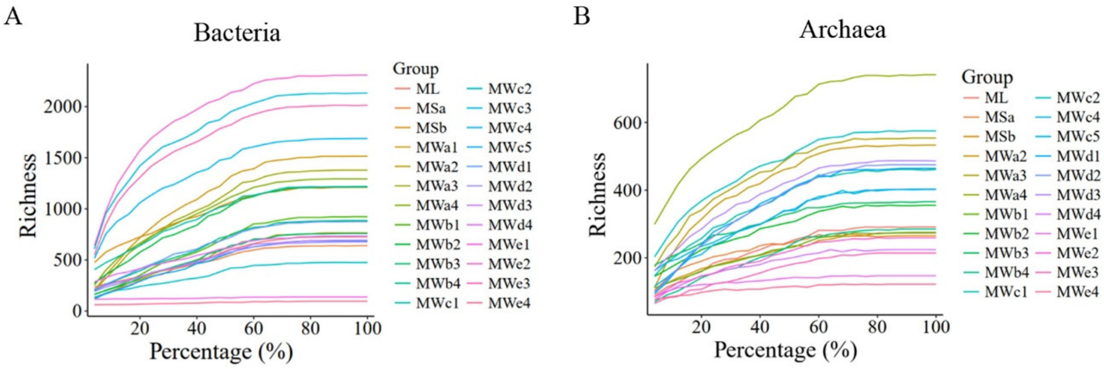

Figure S2. Rarefaction analysis of bacteria (A) and archaea (B) amplicon obtained from different brine samples of the Mahai potash region.

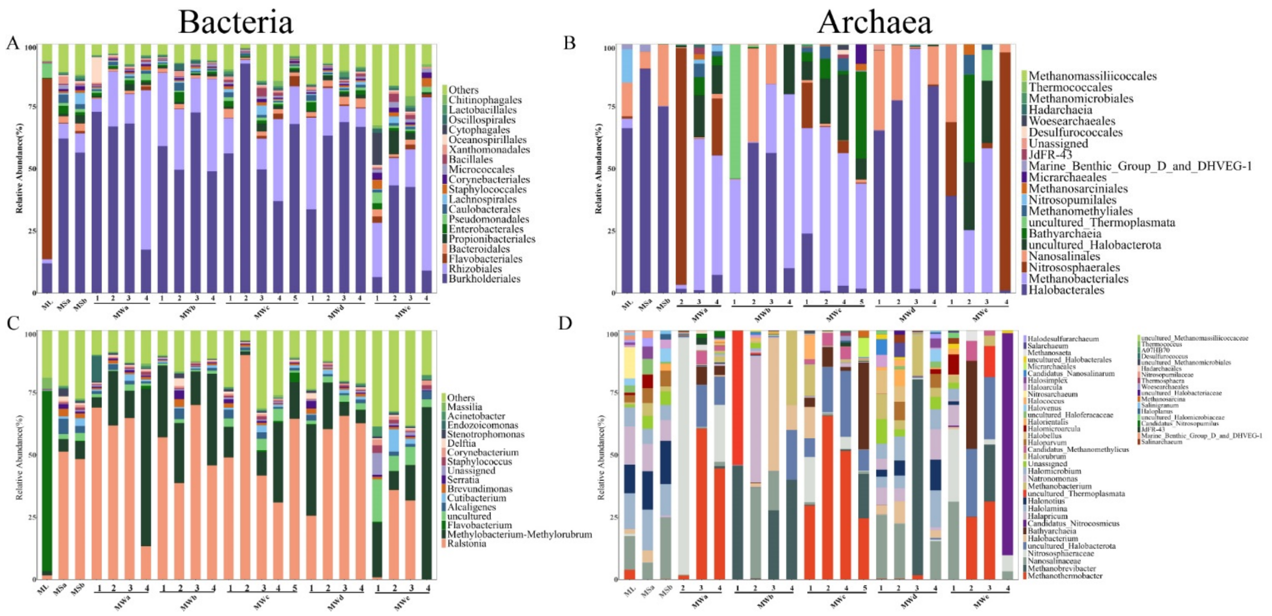

Figure S3. Barplots of bacterial (left) and archaeal (right) orders (A, B) and genera (C, D) detected in the brine samples in Maihai potash area. Relative frequencies correspond to the abundance of bacterial and archaeal groups as percentages of the total identified taxa in each sample. Orders that represented <0.5% of the total bacterial sequences from each assemblage were pooled and depicted as "other".

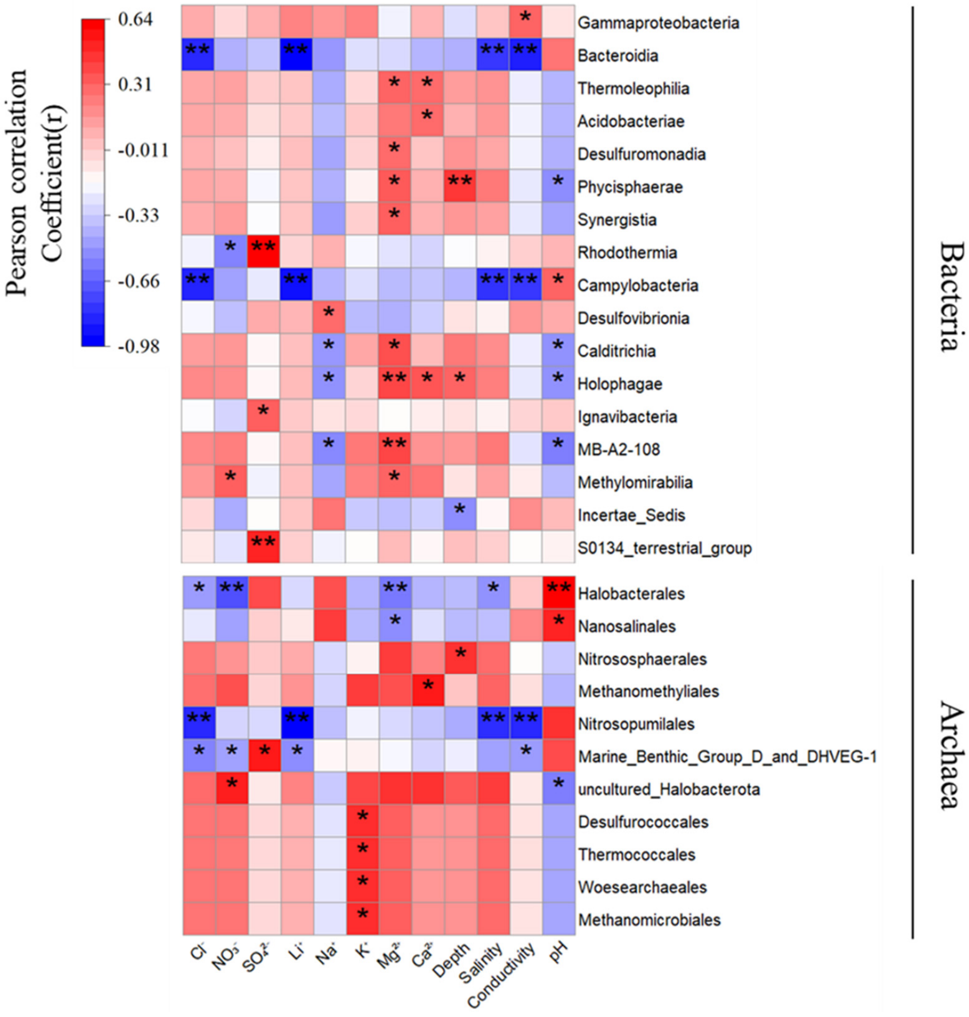

Figure S4. Heatmap of values of Pearson's correlation between species and environmental factors. The heatmap shows the species of bacteria at class level with relative abundance greater than 0.2 % and archaea at order level that are significantly associated with any environmental factor. Lines represent different microbial groups, columns represent different environmental factors. The color of the color block represents the correlation coefficient (r), and red indicates positive correlation, blue negative correlation. If the p value of the correlation between an environmental factor and the abundance of a species is less than 0.05, one star number "*" is marked in the corresponding color block, indicating significant indigenous correlation. If the p <=0.01, two-star numbers "**"are marked, indicating extremely significant indigenous correlation.
